# Sarcomatoid Cholangiocarcinoma of the Common Bile Duct: An Unusual Entity

**DOI:** 10.7759/cureus.37646

**Published:** 2023-04-16

**Authors:** Tatiana Moreira Marques, Gonçalo Ribeiro Miranda, Diogo Melo Pinto, Lilite Barbosa, Gil Faria

**Affiliations:** 1 General Surgery Department, Unidade Local de Saúde de Matosinhos - Hospital Pedro Hispano, Matosinhos, PRT; 2 Pathology Department, Unidade Local de Saúde de Matosinhos - Hospital Pedro Hispano, Matosinhos, PRT

**Keywords:** biliary tumours, jaundice, pancreaticoduodenectomy, cholangiocarcinoma, sarcomatous carcinoma

## Abstract

Sarcomatous carcinoma of the bile duct is tremendously uncommon and aggressive. Herein, we report a case of a male who presented with jaundice. The thoraco-abdominopelvic tomography scan revealed a lesion within the common bile duct highly suspicious of malignancy. After laparoscopic pancreaticoduodenectomy, histological examination revealed a sarcomatous carcinoma. Two years after the initial diagnosis, the patient remains without signs of recurrence. Additional research about this rare condition is needed to improve care and prognosis.

## Introduction

Sarcomatoid carcinoma is a rare malignant entity that is histologically characterized as an epithelial tumor with spindle and/or giant cell areas. It can occur in different locations, but recent data has been showing a tendency for the female genital tract, head, and neck [1]. There are also reports of sarcomatoid carcinomas in the liver, gallbladder, pancreas, and ampulla of Vater [2]. However, it is extremely rare to find a sarcomatoid carcinoma arising from the common bile duct (CBD). There are less than ten cases of sarcomatous choledochal carcinoma (SCC) described in the literature [3,4]. There is one retrospective study with four cases of SCC in which the majority lived for less than two months [4]. Herein, we present a case of SCC in a Caucasian male patient and a review of the literature. 

## Case presentation

A 79-years old male patient presented to the emergency room (ER) with jaundice, choluria, acholia, and pruritus over the course of three weeks. He also complained of abdominal discomfort within the previous two months, mainly in the upper right quadrant, of moderate intensity, without relieving or aggravating factors. In the past medical history, the patient had an appendectomy and an inguinal hernioplasty several years before. He was under statin and antihypertensive medication. On physical examination, he presented icteric sclera and a slightly painful abdomen on the right hypochondrium, with an enlarged and palpable gallbladder.

Laboratory tests revealed abnormal liver enzymes (alkaline phosphatase of 342 U/L [normal range: 40-150 U/L], glutamic oxaloacetate transaminase of 142 U/L [normal range: 5-34 U/L], and gamma-glutamyl transferase of 798 U/L [normal range: <38 U/L]) as well as an elevation of both total (8.24 mg/dL [normal range: 0.2-1.2 mg/dL]) and direct (7.10 mg/dL [normal range: <0.5 mg/dL]) bilirubin levels. The serum levels of carcinoembryonic antigen (CEA) were normal, but carbohydrate antigen (CA) 19-9 levels were increased (45 UI/mL [normal range: <37 UI/mL]).

**Figure 1:**
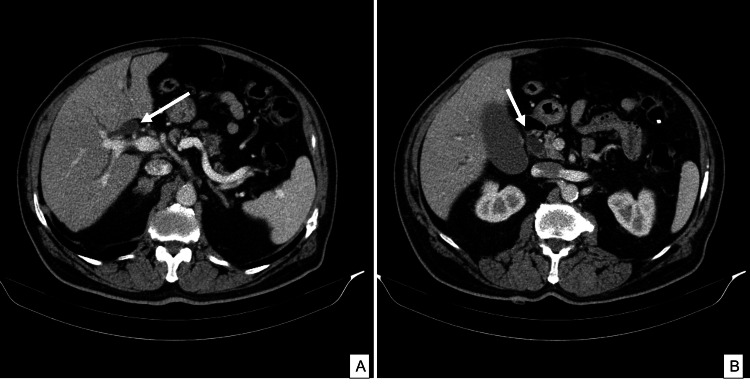
Thoraco-abdominopelvic tomography scan demonstrating enlargement of the common bile duct (A), down to the intrapancreatic portion (B)

Thoraco-abdominopelvic computed tomography (CT) scan diagnosed an enlargement of the common bile duct of 15 mm down to the intrapancreatic portion (Figure 1). The magnetic resonance cholangiopancreatography revealed a sharp dilatation of the bile duct, whose extra-hepatic portion measured a maximum of 22 mm. The transition zone was at the border of the intrapancreatic bile duct, where there was a solid lesion measuring about 12 mm. There were no signs of choledocholithiasis, and no other lesions were reported.

According to protocol at the time, as the patient had a total bilirubin level inferior to 20 mg/dL and was operated within the first week of diagnosis, there was no need to drain the biliary system. Once all was in favor of malignant disease, the multidisciplinary tumor board decided to proceed with surgical treatment. Knowing the importance of complete resection, the patient was submitted to a laparoscopic pancreaticoduodenectomy. The intraoperative frozen section of the proximal margin of the bile duct was negative for neoplastic disease. During the postoperative period, he developed a peripancreatic abscess, drained percutaneously, requiring an extended course of culture-directed antibiotic and antifungal therapies (Escherichia coli and Candida glabrata), but with no evidence of pancreatic fistula. The patient was discharged on the 36th day after surgery.

**Figure 2:**
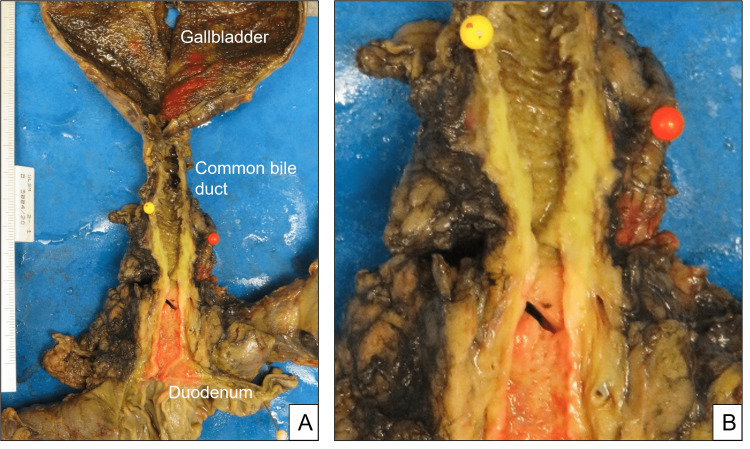
Surgical specimen (A) and common bile duct (B) showing diffuse wall thickening: the tumor is a combined mass-forming and periductal infiltrating type lesion

In the surgical specimen of the pancreaticoduodenectomy (Figure 2A), the common bile duct showed diffuse wall thickening (Figure 2B).

**Figure 3:**
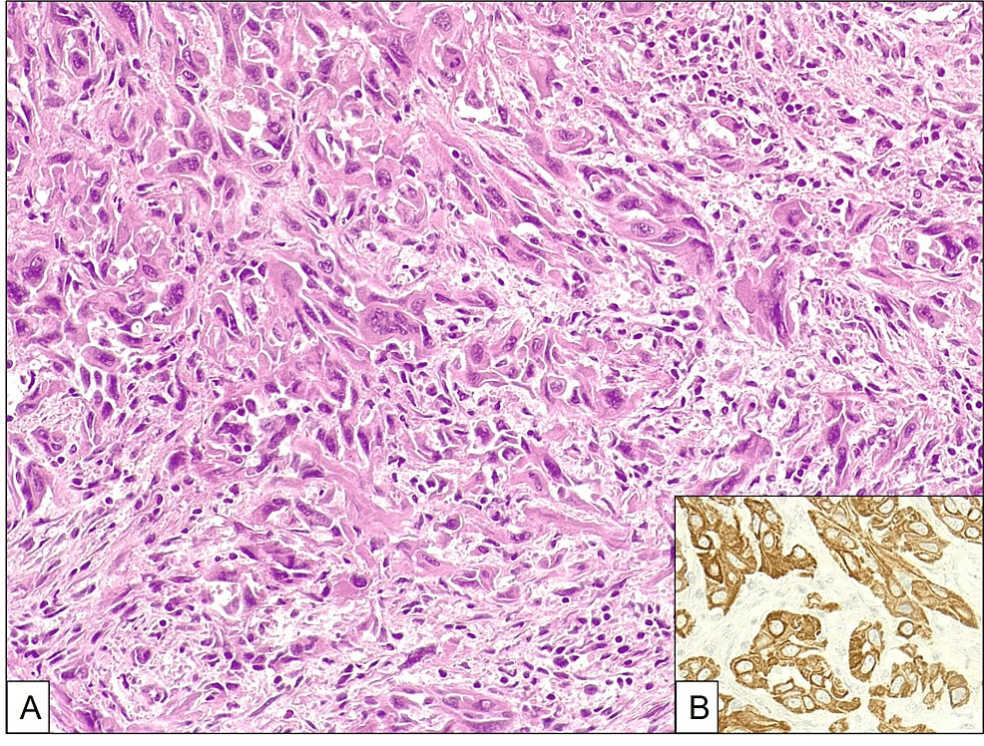
Sarcomatoid carcinoma stained with hematoxylin and eosin The tumor is composed by diffuse sheets of an admixture of spindle and epithelioid cells without heterologous elements with highly pleomorphic nuclei (A). The tumor cells are immunoreactive for cytokeratin 7 (B).

Histologic examination revealed a poorly differentiated carcinoma composed by an admixture of spindle and epithelioid cells with highly pleomorphic nuclei (Figure 3).

No metastases were found in any of the 17 regional lymph nodes (pT2N0M0R0).

At the multidisciplinary tumor board, it was decided to perform clinical surveillance with a six-monthly abdominopelvic CT scan and tumor markers' measurement (CEA and CA 19-9).

Two years after the surgery, the patient remains asymptomatic and with no signs of loco-regional or distant recurrence.

## Discussion

Sarcomatoid carcinoma is an extremely rare tumor that can be found in the hepatobiliary system, namely in the liver [5,6], gallbladder [7], pancreas, and ampulla of Vater [2,8]. Sarcomatoid carcinomas arising in the common bile duct are even scarcer.

It is difficult to differentiate between SCC and more frequent types of choledochal carcinomas, like adenocarcinomas, making the definitive diagnosis based on histopathologic exams of the surgical specimen [4]. There are some radiological features associated with the diagnosis of sarcomatous cholangiocarcinomas. In some studies, they are reported as mixed echoic masses on ultrasonography; however, there are also some studies describing heterogenous hypoechoic masses. On computed tomography scan, it appears as a low-density mass, and in magnetic resonance imaging (MRI), it is described as a hypointense tumor on T1 and a hyperintense tumor on T2 relative to liver parenchyma [4,9].

Concerning SCC's treatment, there is not enough data, and consensus guidelines are far from reality as there are only three case reports available and one retrospective study, which includes four cases, as mentioned above. As for other cholangiocarcinomas, surgical treatment is recommended for resectable tumors. Considering the tumor's site, pancreaticoduodenectomy with lymphadenectomy is the advised procedure [3,4]. Adjuvant chemotherapy is an option; however, more studies are needed to establish a standard treatment, as chemotherapy treatments largely differed between patients/centers from previous reports. Although gemcitabine is suggested in some papers, there is no evidence of its value [4]. 

Zhang et al. have shown that sarcomatous biliary carcinomas, both SCC and intrahepatic cholangiocarcinoma, seem to have a worse prognosis than the more common types of biliary carcinomas, as most patients do not survive over three months. However, there is one patient that survived 12 months, and another one 30 [4]. Yoon et al. proposed that this worse prognosis could be due to diffuse tumor infiltration [10]. However, Jang et al. inferred that tumors located in the CBD, by causing early obstruction of the bile duct with associated symptoms, such as jaundice, could be diagnosed earlier, improving their prognosis [11]. The patient remains recurrence-free 24 months after surgery, which can imply a better prognosis of SCC. It is thought that the pathologic diagnosis of lymph node metastasis and the tumor Ki-67 index can be possible indicators of survival, but more research is required [4].

## Conclusions

We present a case of SCC that was successfully treated with surgical R0 resection, that due to the lack of evidence concerning chemotherapy effectiveness, remains the most important therapy with curative intent. Due to the scarcity of SCC reports, experience from different centers is needed in order to collectively improve the treatment and prognosis of these patients. We consider the utmost importance to report this type of case to gather as much information about this pathology as possible.
